# Case of Internal Hemorrhage, Arising from Rupture of the Fallopian Tube

**Published:** 1824-04

**Authors:** Thomas Bushell

**Affiliations:** Member of the Royal College of Surgeons, and Licentiate of the Society of Apothecaries, London.


Art. V.—*Case of Internal Hemorrhage, arising from Rupture of the Fallopian Tube.* By Mr. Thomas Bushell, Member of the Royal College of Surgeons, and Licentiate of the Society of Apothecaries, London. [Communicated through Dr. James Johnson.]

February 21st, 1824; Saturday, nine o'clock a.m., I was requested to attend, as speedily as possible, upon a female, (Mrs. Garner, of Woodstock-street, New-road,) who was supposed to be dying. On my arrival at her residence, I was informed that she had been seized suddenly with violent pain of the abdomen, about eleven o'clock the preceding night (Friday); she directly fainted, and fell under the fire-grate; but recovering, she went to the adjoining apartment, and called to her assistance a fellow lodger, who placed her on a chair, when she immediately stretched herself, and appeared to be dying. Mrs. G. was put to bed, and continued much in the same state throughout the night, complaining of excessive pain of the lower part of the abdomen; occasionally bedewed with a copious, cold, clammy perspiration; with rigors and fainting. She had several attacks of vomiting, and two fæcal evacuations. Between four and five o'clock *(mane)* she was composed, and dosed for a short time. (A practitioner had been sent for, but had declined visiting her.)

I found her in the following alarming condition: extremely restless; hands and feet quite pale and cold; countenance and lips exsanguine; eyes glassy; pulse hardly perceptible; breathing slightly laborious, and quick; in fact, she seemed to be *in articulo mortis.* She was, however, perfectly sensible; but nearly incapable of speaking, from exhaustion. I applied my hand to her abdomen, suspecting some hernial incarceration, when she cried out, unable to bear the least touch. Considering the poor woman's life would very shortly cease, I apprised the friends; directed warmth to be applied, warm brandy and water to be administered, and sent her a stimulant anodyne draught. Visited her again at half-past ten o'clock: little or alteration; still sensible, and, although with difficulty, she was desirous to express her gratitude for the relief the draught had afforded. She expired about twelve, a period of thirteen hours from the commencement of her sufferings.

Mrs. Garner was of a gay and lively disposition; she had been married between four and five years, had two children, and was forty years of age. Her husband's health and affairs being in a deranged state, she had experienced considerable anxiety of mind, but had stifled her griefs, and was the constant solace of her husband, who was much depressed in spirits. She had not complained of pain or other illness, excepting a trifling occasional uneasiness about the epigastric region. Her husband had left her, about eight o'clock in the evening, in excellent health and spirits; and, on his return about twelve, found her truly moribund.

Permission being obtained to examine the body, about twenty-six hours after her death, I proceeded to the sectio cadaveris; and was accompanied by my friends, Mr. Squibb, of Orchard-street, Mr. Andrews, of Crauford-street, and Mr. Bennet, jun. of Edward-street. The operation was performed by my brother, Mr. R. Bushell, a diligent and sedulous young anatomist.

The abdomen was slightly tumid: on cutting through its parietes, an abundant flow of bloody serum took place; and, from the most moderate calculation, three pints of this fluid must have escaped, and been removed from the peritoneal cavity. Turning upwards the intestines, a large mass of coagulum of blood, at least two pounds, was found in the pelvis; which being withdrawn very cautiously, a gush of about an ounce of yellowish serous fluid esoaped, appearing to come from a circumscribed cavity. The pelvis being quite clear, and the intestines separated, with their mesenteric connexions, the aorta, vena cava, and other blood-vessels, were scrupulously examined; but there was not the least appearance of rupture. The bladder was contracted; the stomach and intestines were healthy, as well as the liver and spleen; but the whole of these viscera were pale. The thoracic organs were natural; the heart loaded with fat.

We now directed our attention to the uterus, which was not larger than in the unimpregnated state. As soon as it was dissected out, the source of the hemorrhage could be readily demonstrated, from a minute ulcerated opening in the back part of the fallopian tube, which was somewhat soft, a little enlarged, and of a livid colour. The point from whence the hemorrhage proceeded being so satisfactory to the gentlemen present, by their advice I took home the uterus and appendages, for more particular investigation.

*Examination of uterus and appendages.*—Uterus not particularly enlarged; probably, however, as much as in the first month of utero-gestation. Peritoneal coat slightly inflamed, or more vascular than in the natural state. Os uteri plugged up by stiff mucus. Slitting up the uterus, the internal surface was turgid, and had a semi-muco-flocculent lining, which was the incipient decidual membrane; also a greater degree of redness at each tubal aperture, more on the right than on the opposite side.

Left fallopian tube, fimbria, and ovarium, quite healthy. Right fallopian tube, for three-fourths of its fimbrial extremity, healthy in structure, but very vascular: inflating it with the blow-pipe, it was pervious, and had every appearance of an ovum having very recently passed through it. The uterine termination enlarged, and nearly twice the size of the left fallopian tube at the same part; and of a dark livid colour, with many turgid vessels ramifying over it. Upon the posterior or sacral aspect of this tumefaction, a small, irregular, jagged hole was visible, either caused by ulceration or sphacelation. This opening would barely admit the pointed extremity of a probe. Making an incision into the dark portion of the uterine extremity of the fallopian tube, a coagulum of blood first made its appearance, and the interior was in some measure disorganised. Dissecting a little more towards the uterus, and deeper through what I have called disorganised, but which, I should rather think, was the shaggy or spongy chorion, a vesicle, about half the size of a pea, was discovered; and, on closer observation, this proved to be the ovum: the minute embryo, certainly not larger than a small pearl-barley, was perfectly distinct, floating in its liquor amnii. To the morsus diaboli of the right fimbria, two or three small hydatiform bodies were attached.

Right ovarium rather larger and more vascular than the left: on cutting into it, a large corpus luteum, occupying nearly one-third of its substance, might be observed. Connected with this ovarium, in its peritoneal duplicature, was a cyst, from which doubtless the fluid rushed out, when scooping away the coagula of blood from the pelvis.

Every thing being so very clear, and as I was disposed to believe, the case fortunately not of very frequent occurrence,—at least, in this early state of the extra-uterine fallopian gestation,—I sent the morbid parts, by my brother, to Dr. James Johnson, for his inspection.

I am well aware that there is nothing very peculiar in the above relation; sufficient, however, to excite attention, whether we consider the small aperture whence the hemorrhage proceeded, or the period of the pregnancy, which I have some reasons for supposing could not have been more than a month advanced. When we reflect on the particular actions which are going on in the uterine system during gestation, and, of course, the great determination of the vital fluid to those important organs at that time, we shall cease to wonder how so large a quantity of blood (five pints) should escape from a pin-hole orifice. The dissection, which I have narrated, demonstrated the uterus and its appendages in rather a high degree of vascularity.

There are two cases of a similar nature, one by Dr. J. Clarke, in the Transactions of a Society, &c. &c., and the other by Mr. Langstaff, in the Medico-Chirurgical Transactions. They are both extremely interesting. In Dr. J. C.'s case, the distention of the tube was nearly the size of a large walnut; the rupture extended about an inch and a half in length; and the calibre of the tube between the fimbria and rupture was rather larger than in the unimpregnated state, which I also observed in the subject of my communication. The hemorrhage amounted to nearly a gallon; the patient dying on the third day from the commencement of the symptoms. The pregnancy was between six and seven weeks advanced.

In Mr. L.'s case, the tube burst in two places; the enlargement was the size of a hen's egg, and two quarts of blood were effused into the cavity of the pelvis. The patient lived seven hours from the first attack.

The symptoms in these cases so nearly resemble those which Mrs. Garner experienced, and the dissections exposed the same facts, (excepting the size of the rupture and tubal enlargement,) that I do not think it necessary to detain your readers longer, but shall quote, as Mr. Langstaff has done, the concluding paragraph of Dr. J. C.'s communication: "Upon the whole, the symptoms which accompanied this disease were such as could not have led any one to form a conjecture respecting the nature of it; and, even if it had been known, we could only have deplored the insufficiency of our art to remedy a situation so uncommon and so fatal."

*117, Crawford-street, Portman-square.*

